# LncRNA WDFY3‐AS2 suppresses proliferation and invasion in oesophageal squamous cell carcinoma by regulating miR‐2355‐5p/SOCS2 axis

**DOI:** 10.1111/jcmm.15488

**Published:** 2020-06-14

**Authors:** Qing Zhang, Fangxia Guan, Tianli Fan, Shenglei Li, Shanshan Ma, Yanting Zhang, Wenna Guo, Hongtao Liu

**Affiliations:** ^1^ School of Life Sciences Zhengzhou University Zhengzhou China; ^2^ Department of Pharmacology School of Basic Medicine Zhengzhou University Zhengzhou China; ^3^ Department of Pathology The First Affiliated Hospital of Zhengzhou University Zhengzhou China

**Keywords:** ceRNA mechanism, invasion and metastasis, microRNA‐2355‐5p, oesophageal squamous cell carcinoma, suppressors of cytokine signalling 2, WDFY3‐AS2

## Abstract

Long non‐coding RNAs (lncRNAs) widely participate in ESCC development and progression; however, the prognostic factors and therapeutic strategies implicated in ESCC development and progression remain to be under investigation. The purpose of the current study was to explore whether WDFY3‐AS2 may be a potential prognostic factor and investigate its biological functions in ESCC. Here, WDFY3‐AS2 was frequently down‐regulated in ESCC tissues and cells, and its expression was correlated with TNM stage, lymph node metastasis and poor prognosis of ESCC patients. Moreover, WDFY3‐AS2 down‐regulation significantly promoted cell proliferation and invasion, whereas WDFY3‐AS2 up‐regulation markedly suppressed cell proliferation and invasion in ESCC EC9706 and TE1 cells, coupled with EMT phenotype alterations. WDFY3‐AS2 functioned as a competing endogenous RNA (ceRNA) for sponging miR‐2355‐5p, further resulted in the up‐regulation of its target gene SOCS2, followed by suppression of JAK2/Stat5 signalling pathway, to suppress ESCC cell proliferation and invasion in EC9706 and TE1 cells. These findings suggest that WDFY3‐AS2 may participate in ESCC development and progression, and may be a novel prognostic factor for ESCC patients, and thus targeting WDFY3‐AS2/miR‐2355‐5p/SOCS2 signalling axis may be a novel therapeutic strategy for ESCC patients.

## 1 INTRODUCTION

Oesophageal squamous cell carcinoma (ESCC) as one of the most common occurring digestive tumours in the world mainly distributes in Asian and African areas.^1^ Currently, although tremendous progression in the diagnosis and therapy of ESCC, the 5‐year survival rate of ESCC patients remains quite poor,^2^, ^3^ which may be closely associated with diagnosis at an advanced stage and lymph node metastasis.^4^, ^5^ With the development of sequencing technology, long non‐coding RNAs (lncRNAs) have been developed as novel diagnosis and prognosis biomarkers for many different tumours,^6^ which will provide the novel insights into the mechanisms of ESCC development and progression.^7^ Therefore, it is important to seek for novel lncRNAs as the diagnostic and prognostic factors for ESCC patients to improve the prognosis of ESCC patients.

LncRNAs are the class of RNA molecules harbouring more than 200 nucleotides, without protein‐coding potential.^8^ At present, lncRNAs are considered to play important regulatory roles in the transcriptional and post‐transcriptional levels,^9^ further eliciting many important regulatory networks in multiple different tumours.^10^, ^11^, ^12^ Whelming evidence has revealed that lncRNAs are widely implicated in the regulation of cell growth, survival, differentiation, cell cycle, apoptosis, invasion and metastasis,^13^, ^14^, ^15^, ^16^, ^17^ which will promote or suppress tumour development and progression, mainly depending on onco‐lncRNAs or tumour suppressor lncRNAs.^18^ Our previous report revealed differential expression lncRNAs in oesophageal cancer (ESCA), in which WDFY3‐AS2 was tightly associated with prognosis of ESCA patients,^19^ which will be given our exclusive attention for further investigation for its function in ESCC. Recent investigation unveiled that WDFY3‐AS2 suppressed ovarian cancer progression by sponging miR‐18a^20^ and WDFY3‐AS2 may be a novel valuable prognostic factor for the patients with diffuse glioma.^21^ These findings highlight the role of WDFY3‐AS2 in tumour development and progression and will provide the deep insight into the underlying molecular mechanisms of WDFY3‐AS2 in many other type tumours in future.

In the current study, we showed that low WDFY3‐AS2 expression was tightly associated with TNM stage, lymph node metastasis and poor prognosis of ESCC patients. WDFY3‐AS2 down‐regulation significantly promoted cell proliferation and invasion, whereas WDFY3‐AS2 up‐regulation markedly suppressed cell proliferation and invasion in ESCC EC9706 and TE1 cells, coupled with EMT phenotype alterations. Mechanistically, WDFY3‐AS2 promoted the expression of suppressors of cytokine signalling 2 (SOCS2) by sponging miR‐2355‐5p and further triggered the inactivation of JAK2/Stat5 signalling pathway, which resulted in the suppression of cell proliferation and invasion of ESCC cells. Taken altogether, these findings reveal the clinical significance of WDFY3‐AS2 and the essential roles of WDFY3‐AS2/miR‐2355‐5p/SOCS2 signalling axis in ESCC development and progression.

## 2 MATERIALS AND METHODS

### 2.1 Tissue samples

Forty‐five cases of ESCC samples and paired normal oesophageal epithelial tissues were obtained from the First Affiliated Hospital of Zhengzhou University from 2010 to 2016. Tissue samples were confirmed using H&E staining by experienced pathologists from the First Affiliated Hospital of Zhengzhou University. Informed consent of all tissue samples was obtained from each patient, and the utilization of tissue samples was approved by the Research and Ethics Committee of our hospital in this study.

### 2.2 Public database assay

GEO DataSet (GSE111011) was employed for WDFY3‐AS2 expression in 7 cases of ESCC patients and 7 cases of paired normal samples. The Cancer Genome Atlas (TCGA) database from ENCORI (The Encyclopedia of RNA Interactomes) was used for WDFY3‐AS2 and miR‐2355‐5p expression in 162 cases of ESCA patients and 11 cases of normal samples as well as the other type tumours. LncATLAS online software (http://lncatlas.crg.eu/) was utilized to predict its subcellular localization of WDFY3‐AS2.

### 2.3 Cell lines, cell culture and transfection

Human ESCC cell lines including Eca109, EC9706, TE1, KYSE140, KYSE520, KYSE150 and KYSE180 as well as normal oesophageal epithelial cell Het‐1A were obtained from the Chinese Academy of Sciences Cell Bank, which was maintained in RMPI 1640 medium supplemented with 10% Foetal Bovine Serum (Gibco, Invitrogen, USA) in a humidified incubator harbouring 5% CO_2_. WDFY3‐AS2 siRNA #1, 2 and 3, si‐Ctrl, (Table S1), pcDNA3.1, pcDNA3.1‐WDFY3‐AS2 (Table S2), miR‐2355‐5p mimic, miR‐2355‐5p inhibitor, negative control (GenePharma, Shanghai, China), SOCS2 siRNA, control siRNA (Santa Cruz company, USA) and pcDNA3.1‐SOCS2 (Table S2) were transfected into EC9706 and TE1 cells by Lipofectamine^™^ 2000 (Invitrogen Life Technologies, Carslbad, CA, USA) according to manufacturer's instruction.

### 2.4 CCK‐8 assay for cell proliferation

EC9706 and TE1 cells were transfected with WDFY3‐AS2 siRNA, si‐Ctr1, pcDNA3.1, pcDNA3.1‐WDFY3‐AS2, miR‐2355‐5p mimic, miR‐2355‐5p inhibitor, negative control, SOCS2 siRNA, control siRNA and pcDNA3.1‐SOCS2 in triplicate according to manufacturer's protocol, and then, various cells (approximate 2000 cells/ well) were seeded into 96‐well plate. At the time of measurement, CCK‐8 (Beyotime Biotech, Haimen, China) was added to corresponding wells, and absorbance value at 450 nm was determined in a microplate reader (Thermo Scientific, Waltham, MA).

### 2.5 EdU staining assay

EdU staining assay was performed according to manufacturer's protocol. EC9706 and TE1 cells (5 × 10^5^ cells/well) were seeded into 24‐well plate, and then were transfected with si‐Ctrl, WDFY3‐AS2 siRNA, pcDNA3.1 and pcDNA3.1‐WDFY3‐AS2 according to manufacturer's protocol. Cells were labelled with EdU reagent in a final concentration of 50 μmol/L for 2 hours. Cells were rinsed using PBS buffer for 5 minutes. Subsequently, cells were fixed using PBS buffer containing 4% polyformaldehyde for 30 minutes, and glycine (2 mg/mL) in a volume of 50 μL was added to ESCC cells for 5 minutes. Finally, TritonX‐100 in a volume of 100 μL was used for decolorization for 10 minutes. Regarding Apollo staining, a total of 100 μL of 1× Apollo staining liquid was applied to each well and incubated for 30 minutes. PBS buffer containing 0.5% TritonX‐100 in a volume of 100 μL was used for decolorization for 10 minutes. Finally, DNA staining was performed using DAPI according to manufacturer's instruction. The photograph was taken using fluorescent microscope.

### 2.6 Invasion experiment by Transwell chamber

Cell invasion was investigated by Transwell chamber harbouring Matrigel (BD Biosciences, San Diego, CA, USA) according to previous report.^22^ Briefly, EC9706 and TE1 cells (1 × 10^5^) were placed in the upper layer of chamber; meanwhile, 20% FBS was added to underlayer of chamber. Subsequently, invasive cells were fixed using methanol, followed by staining with crystal violet 48 hours after transfection. Finally, the number of invasive cells was investigated under the field of 200× magnification.

### 2.7 Subcellular fractionation

Cell nucleus and cytoplasm RNA isolation kit (Beibei, Biotech, Co.Ltd, China) was used to extract the nuclear RNA and cytoplasmic RNA, respectively, according to manufacturer's instruction, and then investigated using qRT‐PCR (Table S3).

### 2.8 *Fluorescence* in situ* hybridization (FISH)*

For FISH assay, EC9706 and TE1 cells were grown in 24‐well plates with glass coverslips for 24 hours. After immobilization and permeabilization, EC9706 and TE1 cells were hybridized with 20 μmol/L Cy3‐labelled WDFY3‐AS2 probe (RiboBio), and 6‐diamidino‐2‐phenylindole (DAPI) was used to stain nuclei. The images were observed with a florescent microscope.

### 2.9 Real‐time quantitative PCR (qRT‐PCR)

Total RNA was isolated by Trizol (Invitrogen) according to the manufacturer's instructions. For mRNA analysis, qRT‐PCR was performed using Power SYBR_ green PCR master mix (Applied Biosystems) on an ABI 7500 series PCR machine Applied Biosystems using the specific primers (Table S3). For miR‐2355‐5p expression assay, total RNA was reverse transcribed using the miScript Reverse Transcription Kit (Qiagen, Valencia, CA). qRT‐PCR amplification for miR‐2355‐5p was performed using the miScript PCR Kit (Qiagen) using the specific primers(Table S3). Experiments were normalized to U6.

### 2.10 Western blot

Total proteins were extracted from ESCC cells using RIPA lysis (Solarbio, Beijing, China), and the concentration was measured by Bradford method. The proteins were separated by SDS‐PAGE and then transferred to PVDF membranes (Millipore Corporation, Bedford, MA, USA). The primary antibodies against E‐cadherin, N‐cadherin, Vimentin, Stat5, p‐Stat5, JAK2, p‐JAK2, SOCS2 and β‐actin (1:200 dilution, Abcam, Cambridge, MA, USA) were incubated with PVDF membrane (Roche, Switzerland) overnight at room temperature after blocking with skimmed milk. Subsequently, the secondary antibody (ZSGB‐BIO, Guangzhou, China) was added to PVDF membrane. Finally, enhanced chemiluminescence (ECL) reagents (Beyotime, Haimen, China) were utilized to develop the protein signal.

### 2.11 Dual‐luciferase reporter assay

The dual‐luciferase reporter assay system was conducted to determine the direct target of WDFY3‐AS2 and miR‐2355‐5p. Recombinant vector pmirGLO‐WDFY3‐AS2‐WT and pmirGLO‐WDFY3‐AS2‐MUT as well as pmirGLO‐SOCS2‐WT and pmirGLO‐SOCS2‐MUT (TSINGKE Biological Technology, Beijing, China) along with miR‐2355‐5p mimic and negative control (NC) were transfected into EC9706 and TE1 cells, respectively. Luciferase activity was determined using the Dual‐Luciferase Reporter Assay System (Promega, USA) 48 hours after transfection according to manufacturer's instruction.

### 2.12 RNA immunoprecipitation (RIP)

RIP assay was performed in ESCC EC9706 and TE1 cells by using RNA‐binding protein immunoprecipitation kit (Millipore, Billerica, MA, USA) as described previously.^23^, ^24^ Briefly, RIP lysates were prepared from EC9706 and TE1 cells transfected with miR‐2355‐5p mimic or NC, and then were subjected to immunoprecipitation using 5 μL of either a normal mouse IgG or 5 μL of Anti‐Ago2 antibody and the Mana RIP^™^ RNA‐binding Protein Immunoprecipitation Kit. The mRNA levels of WDFY3‐AS2 and miR‐2355‐5p enriched on beads were determined by qRT‐PCR (Table S3). The expressions of all other miRNAs in Table S4 were detected using the corresponding NCBI database sequences as forward primers.

### 2.13 Statistical assay

All experimental data were investigated using GraphPad Prism 6.0 software. Data were presented as mean with standard deviation (SD). The survival assay was performed using log‐rank test. The comparisons of two groups were determined using *t* test, and comparisons of three groups or above were investigated using one‐way ANOVA. A *P* value less than .05 was regarded to be significant.

## 3 RESULTS

### 3.1 WDFY3‐AS2 is down‐regulated in ESCC and associated with poor prognosis

**Figure 1:**
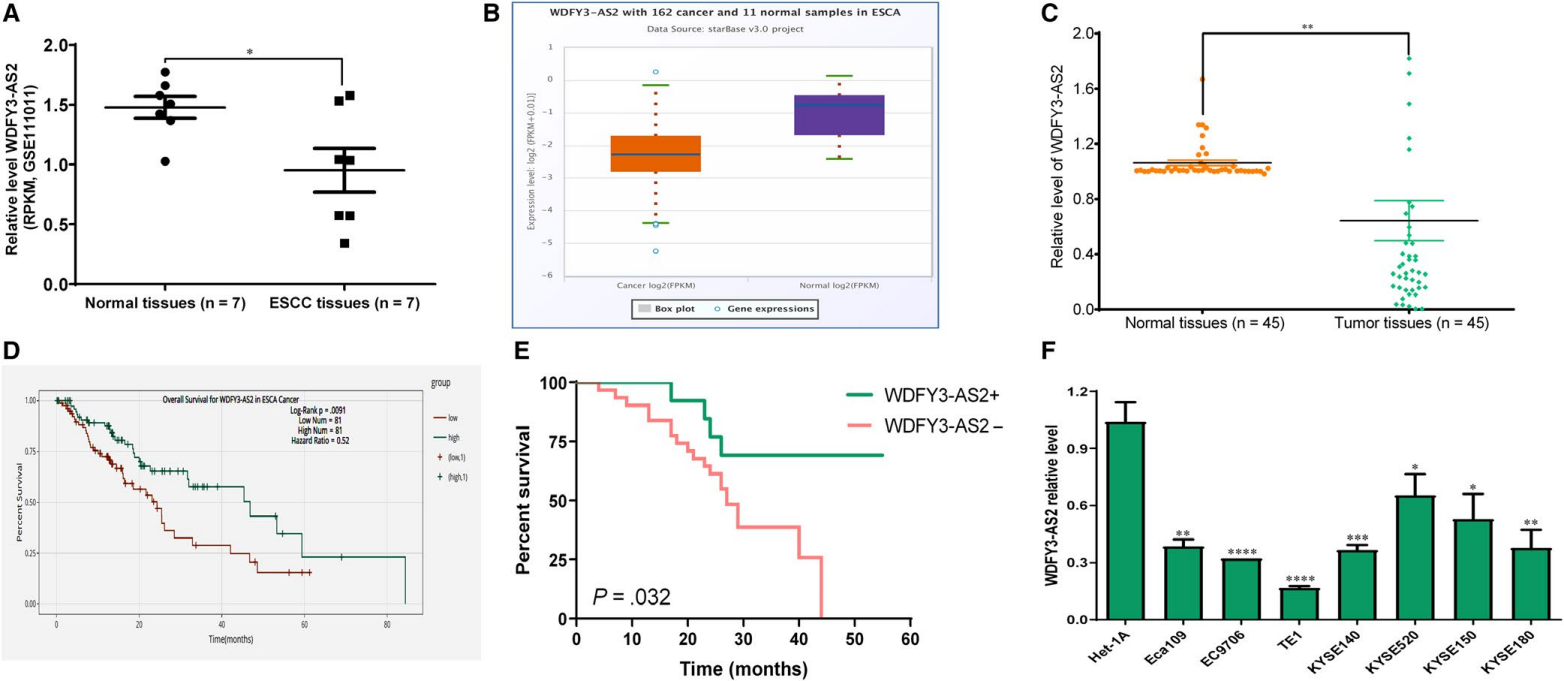
Decreased WDFY3‐AS2 level was tightly associated with the prognosis of ESCC patients. A. GEO Dataset GSE11011 assay for WDFY3‐AS2 level in ESCC tissues and paired normal oesophageal tissues; B. TCGA database assay for WDFY3‐AS2 level in ESCA tissues and paired normal oesophageal tissues; C. qRT‐PCR assay for WDFY3‐AS2 level in 45 cases of ESCC tissues and paired normal oesophageal tissues; D. TCGA assay for the correlations between WDFY3‐AS2 expression and ESCA patients’ prognosis; E. Log‐rank test assay for association of WDFY3‐AS2 level with ESCC patients’ survival time; F. Expression of WDFY3‐AS2 in a panel of ESCC cells including Eca109, EC9706, TE1, KYSE140, KYSE520, KYSE150 and KYSE180 as well as normal oesophageal epithelial cell Het‐1A. *P < .05, **P < .01, ***P < .001 and ****P < .0001, compared with normal tissues or normal oesophageal epithelial cell Het‐1A

Our previous study has demonstrated that WDFY3‐AS2 is predicted to be down‐regulated in ESCA and may be a novel prognostic factor of ESCA.^19^ To investigate the underlying functions of WDFY3‐AS2 in ESCC, GEO DataSets and TCGA database were employed to investigate the expressions of WDFY3‐AS2 in ESCA tissues. We found that WDFY3‐AS2 expression in ESCA tissues was significantly lower than that in normal oesophageal tissues (*P* < .05) (Figure 1A and B), which was further confirmed by qRT‐PCR in 45 cases of ESCC tissues and paired normal tissues (Figure 1C). Stepwise investigation from StarBase software revealed that the overall survival rate of ESCA patients with high WDFY3‐AS2 was higher than those with low WDFY3‐AS2 (*P* < .01) (Figure 1D), to validate this result, log‐rank test was utilized to detect the correlations between WDFY3‐AS2 and the survival rate of ESCC patients, and the same results were obtained in Figure 1E. Besides, the expression of WDFY3‐AS2 in all ESCC cells was markedly lower than that in normal oesophageal epithelial cell Het‐1A (Figure 1F). Moreover, the investigation about RNA sequencing data derived from TCGA demonstrated that WDFY3‐AS2 was markedly down‐regulated in fifteen other different types of tumours (Figure S1). Most notably, low WDFY3‐AS2 level was tightly associated with poor prognosis of five various types of tumours (Figure S2). These data presented herein indicate that low WDFY3‐AS2 level is a frequent occurring event in multiple tumour types, including ESCC, and may be a novel prognostic factor for multiple different types of tumours, especially for ESCC patients.

### 3.2 WDFY3‐AS2 is correlated with TNM stage and lymph node metastasis in ESCC

**Figure 2:**
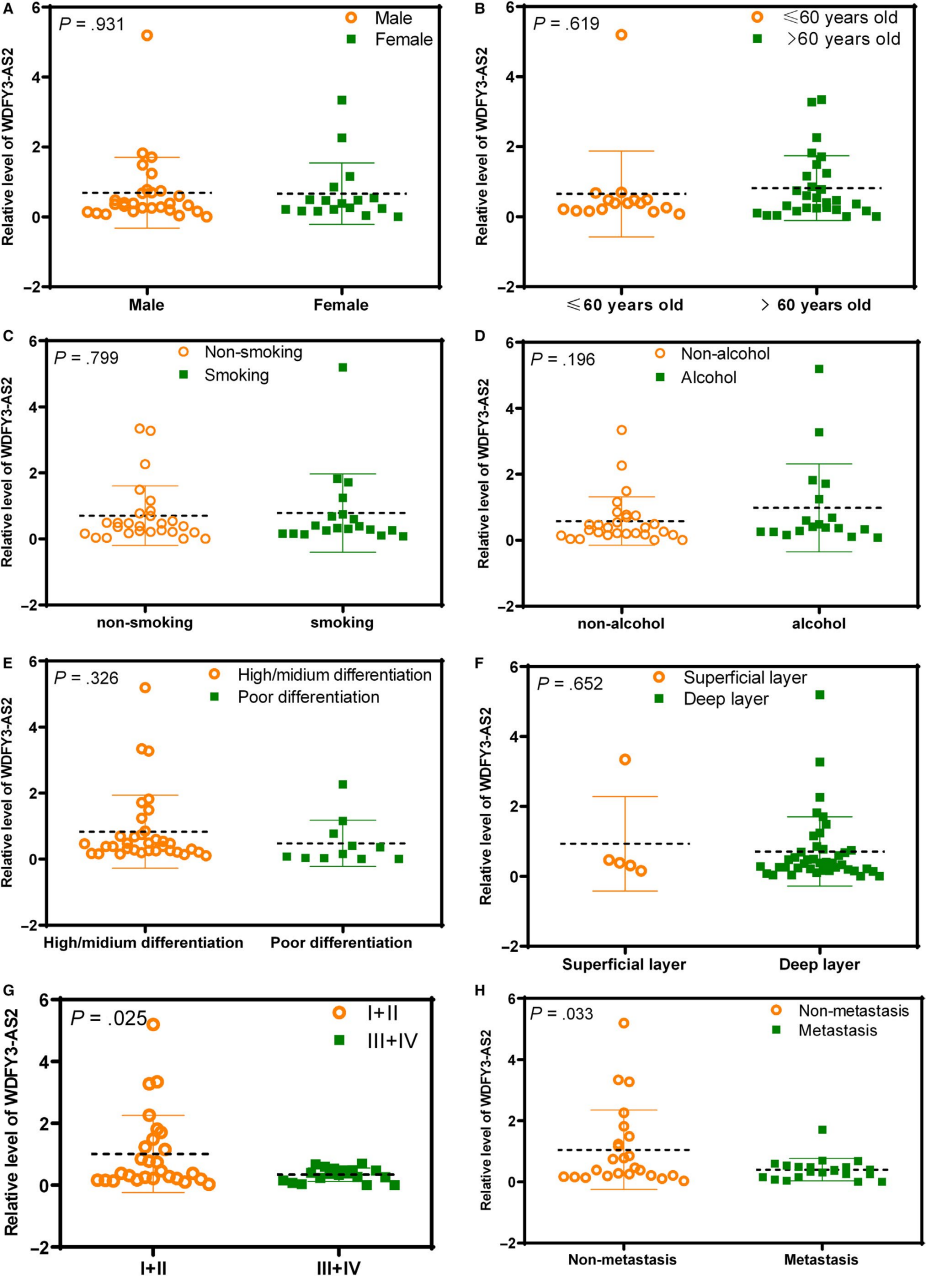
WDFY3‐AS2 is associated with clinicopathological features in ESCC. qRT‐PCR was used to detect the WDFY3‐AS2 level in ESCC tissues, and data were statistically analysed using GraphPad Prism 6.0 software; a *P* value less than .05 was considered as statistical significance

To further dissect the association of WDFY3‐AS2 expression with ESCC development and progression, GraphPad software was used to investigate the correlations of WDFY3‐AS2 expression with clinicopathological features such as gender, age, smoking, alcohol, tumour differentiation, invasion depth, TNM stage and lymph node metastasis. The results demonstrated that WDFY3‐AS2 expression was closely associated with TNM stage and lymph node metastasis, but not related to the patients’ gender, age, smoking, alcohol, tumour differentiation and invasion depth (Figure 2A‐H). These findings suggest that WDFY3‐AS2 may participate in ESCC development and progression.

### 3.3 WDFY3‐AS2 suppresses cell proliferation and invasion in ESCC cells

**Figure 3:**
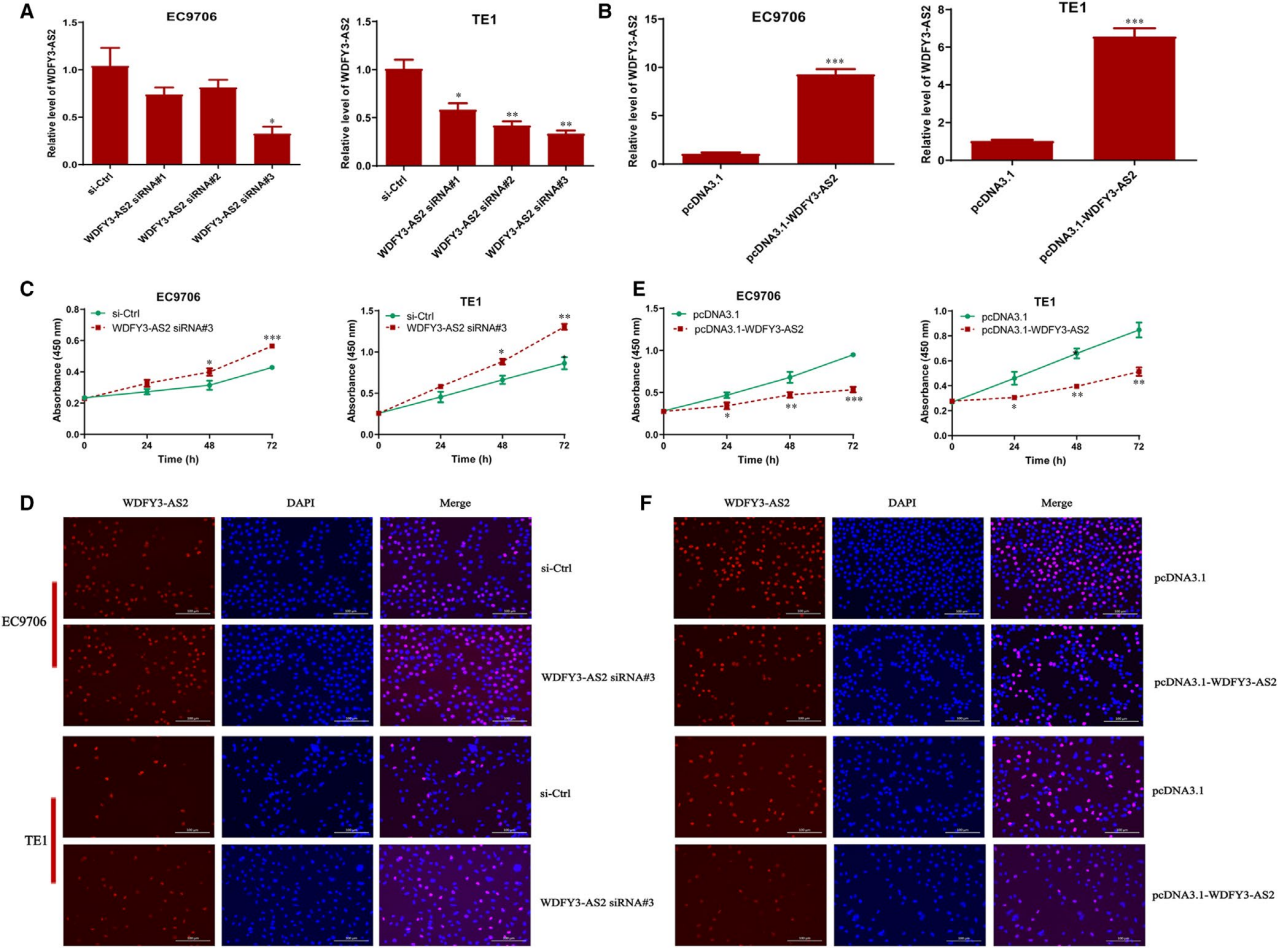
WDFY3‐AS2 suppresses cell proliferation in ESCC cells. A. qRT‐PCR assay for WDFY3‐AS2 expression after transfection with WDFY3‐AS2 siRNA and si‐Ctrl in EC9706 and TE1 cells; B. qRT‐PCR assay for WDFY3‐AS2 expression after transfection with pcDNA3.1‐WDFY3‐AS2 and control plasmid pcDNA3.1 in EC9706 and TE1 cells; C. CCK‐8 detection for cell proliferation in EC9706 and TE1 cells transfected with WDFY3‐AS2 siRNA and si‐Ctrl; D. EdU staining assay for cell proliferation in EC9706 and TE1 cells transfected with WDFY3‐AS2 siRNA and si‐Ctrl, Bar=100μm; E. CCK‐8 detection for cell proliferation in EC9706 and TE1 cells transfected with pcDNA3.1‐WDFY3‐AS2 and pcDNA3.1; F. EdU staining assay for cell proliferation in EC9706 and TE1 cells transfected with pcDNA3.1‐WDFY3‐AS2 and pcDNA3.1, Bar=100μm. The experimental data were obtained from independently repeated at least three times, *P < .05, **P < .01 and ***P < .001, compared with si‐Ctr1 or pcDNA3.1 treatment group

**Figure 4:**
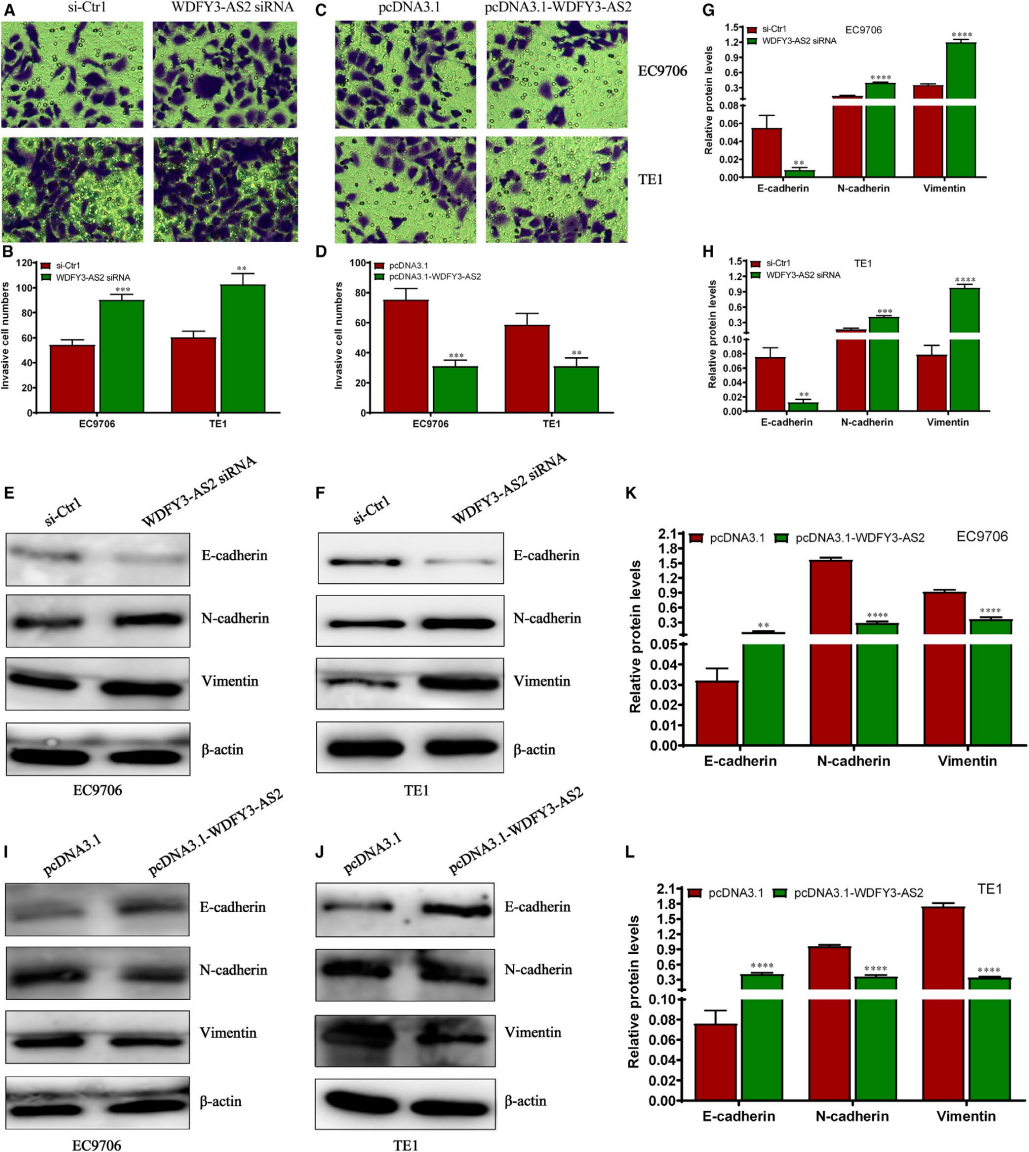
WDFY3‐AS2 suppresses cell invasion and EMT process in ESCC cells. A. Transwell assay for cell invasion following transfection with WDFY3‐AS2 siRNA and si‐Ctrl in EC9706 and TE1 cells; B. Statistical assay of invasive cell numbers in EC9706 and TE1 cells after treatment with WDFY3‐AS2 siRNA and si‐Ctrl; C. Transwell assay for cell invasion after transfection with pcDNA3.1‐WDFY3‐AS2 and pcDNA3.1 in EC9706 and TE1 cells; D. Statistical assay of invasive cell numbers in EC9706 and TE1 cells after treatment with pcDNA3.1‐WDFY3‐AS2 and pcDNA3.1; E and F. Western blot detection for the expressions of E‐cadherin, N‐cadherin and Vimentin proteins in EC9706 and TE1 cells after transfection with WDFY3‐AS2 siRNA and si‐Ctrl; G. Relative levels of E‐cadherin, N‐cadherin and Vimentin proteins in EC9706 cells after transfection with WDFY3‐AS2 siRNA and si‐Ctrl; H. Relative levels of E‐cadherin, N‐cadherin and Vimentin proteins in TE1 cells after transfection with WDFY3‐AS2 siRNA and si‐Ctrl; I and J. Western blot detection for the expressions of E‐cadherin, N‐cadherin and Vimentin proteins in EC9706 and TE1 cells after transfection with pcDNA3.1‐WDFY3‐AS2 and pcDNA3.1; K. Relative levels of E‐cadherin, N‐cadherin and Vimentin proteins in EC9706 cells after transfection with pcDNA3.1‐WDFY3‐AS2 and pcDNA3.1; L. Relative levels of E‐cadherin, N‐cadherin and Vimentin proteins in TE1 cells after transfection with pcDNA3.1‐WDFY3‐AS2 and pcDNA3.1. The experimental data were obtained from independently repeated at least three times, **P< .01, ***P < .001 and ****P < .0001, compared with si‐Ctr1 or pcDNA3.1 treatment group

To unveil the underlying biological roles of WDFY3‐AS2 in ESCC progression, three different siRNAs against WDFY3‐AS2 (WDFY3‐AS2 siRNA#1, #2 and #3) and a WDFY3‐AS2‐overexpressing plasmid (pcDNA3.1‐WDFY3‐AS2) were transfected into EC9706 and TE1 cells, and qRT‐PCR was utilized to confirm the transfection efficiency. We found that WDFY3‐AS2 siRNA #3 significantly reduced WDFY3‐AS2 level in EC9706 and TE1 cells (Figure 3A), whereas pcDNA3.1‐WDFY3‐AS2 markedly promoted WDFY3‐AS2 expression in EC9706 and TE1 cells (Figure 3B). CCK‐8 and EdU staining results exhibited obvious proliferation‐promotion efficacy in EC9706 and TE1 cells transfected with WDFY3‐AS2 siRNA, compared to those transfected with si‐Ctrl (Figure 3C and D), whereas the opposite data were obtained after WDFY3‐AS2 overexpression (Figure 3E and F). To further explore the role of WDFY3‐AS2 in ESCC cell invasion, Transwell chamber was employed to investigate cell invasion in different transfection ESCC cells. The current data revealed that WDFY3‐AS2 silencing significantly promoted cell invasion (Figure 4A and B); in contrast, WDFY3‐AS2 overexpression markedly suppressed cell invasion (Figure 4 C and D). To further dissect the potential mechanism, the expressions of EMT‐related proteins such as E‐cadherin, N‐cadherin and Vimentin were investigated by Western blot. The results revealed that WDFY3‐AS2 down‐regulation reduced E‐cadherin level, but enhanced the levels of N‐cadherin and Vimentin proteins (Figure 4E‐H); however, WDFY3‐AS2 up‐regulation promoted E‐cadherin level and suppressed the levels of N‐cadherin and Vimentin proteins (Figure 4I‐L). These data indicate that WDFY3‐AS2 functions as a tumour suppressor in ESCC and its involvement in the regulation of cell invasion may be associated with EMT phenotype in ESCC cells.

### 3.4 WDFY3‐AS2 regulates miR‐2355‐5p level by acting as a ceRNA

**Figure 5:**
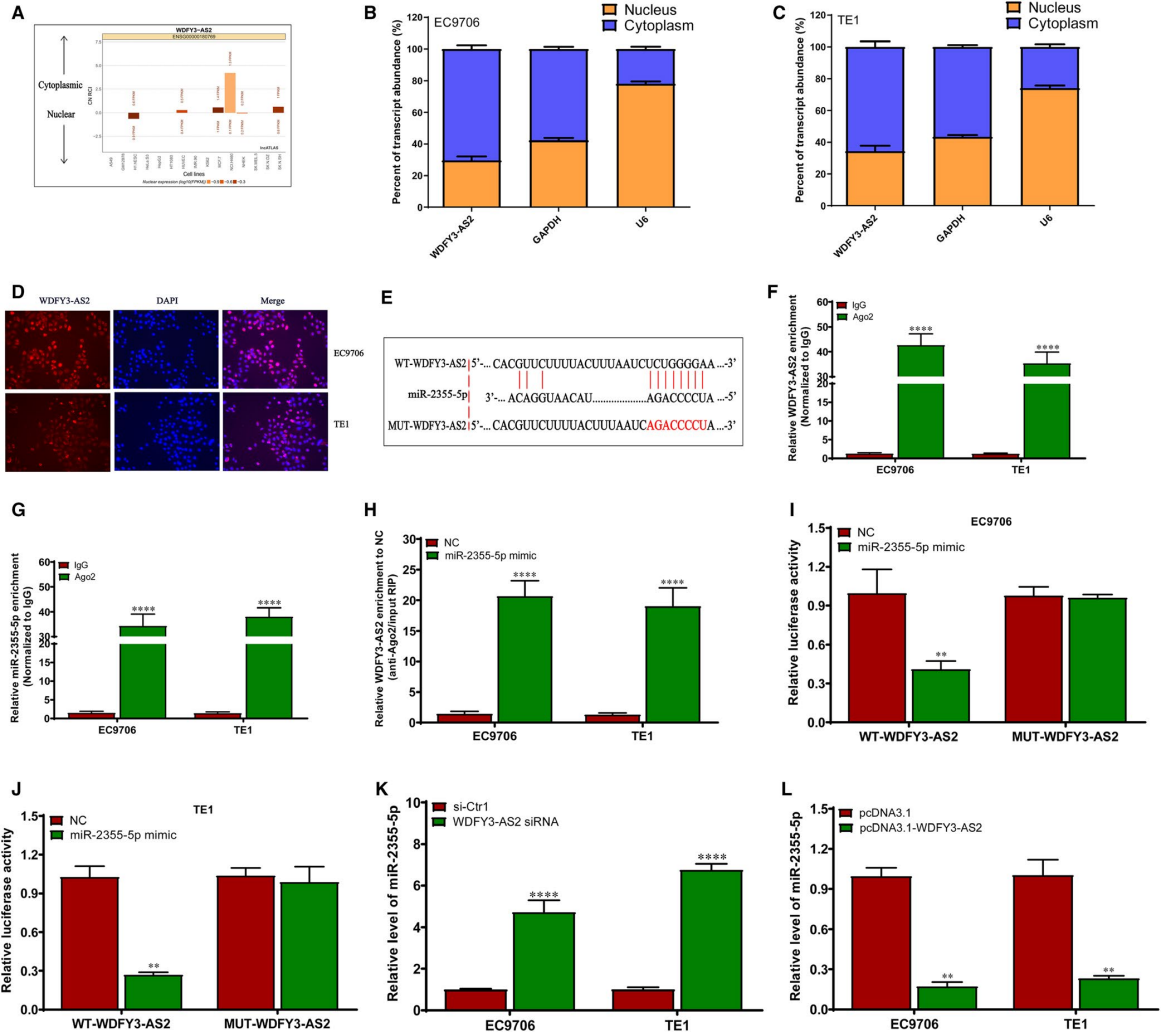
WDFY3‐AS2 sponges miR‐2355‐5p by acting as a ceRNA. A. The localization of WDFY3‐AS2 was predicted by the bioinformatics online software lncATLAS; B‐C. qRT‐PCR assay for subcellular WDFY3‐AS2 expression in the nucleus and cytoplasm of EC9706 and TE1 cells, GAPDH and U6 were used as endogenous control; D. FISH assay for subcellular localization of WDFY3‐AS2 in EC9706 and TE1 cells. WDFY3‐AS2 was stained red (Cy3), and nuclei was stained blue (DAPI); E. The predicted miR‐2355‐5p binding sites in the WDFY3‐AS2 transcript; F. Relative enrichment of WDFY3‐AS2 in RIP using anti‐Ago2 antibody in EC9706 and TE1 cells, and the fold enrichment of WDFY3‐AS2 normalized to IgG as negative control; G. Relative enrichment of miR‐2355‐5p in RIP using anti‐Ago2 antibody in EC9706 and TE1 cells, and the fold enrichment of miR‐2355‐5p normalized to IgG as negative control; H. Relative enrichment of WDFY3‐AS2 in EC9706 and TE1 cells transfected with miR‐2355‐5p mimic or NC; I and J. The luciferase activity assay in EC9706 and TE1 cells cotransfected with wide‐type (WT) or mutant WDFY3‐AS2 plasmid together with miR‐2355‐5p mimic or NC;K. qRT‐PCR assay for miR‐2355‐5p level in EC9706 and TE1 cells transfected with WDFY3‐AS2 siRNA or si‐Ctr1; L. qRT‐PCR assay for miR‐2355‐5p level in EC9706 and TE1 cells transfected with pcDNA3.1‐WDFY3‐AS2 or pcDNA3.1. **P < .01 and ****P < .0001 were considered as statistical significance. The experimental data, expressed as mean ± SD, were obtained from three independent repeats

It is well documented that lncRNAs interact with miRNAs by ceRNA, which further manipulates the expression of downstream genes.^25^, ^26^, ^27^, ^28^, ^29^ To explore the underlying molecular mechanisms of WDFY3‐AS2 in ESCC, lncATLAS online software was utilized to predict its subcellular localization. We found that WDFY3‐AS2 was predicted to be mainly appeared in the cytoplasm of the indicated cell types (Figure 5A). qRT‐PCR assay revealed that WDFY3‐AS2 was mainly found in the cytoplasm of EC9706 and TE1 cells (Figure 5B and C), which was confirmed by FISH experiment (Figure 5D). According to its localization, WDFY3‐AS2 may function as a ceRNA. LncBase Predicted v.2 integrated into DIANA Tools was used to predict the possible binding miRNAs, and we selected the top 25 miRNAs for further assay (Table S4). Further investigation from Ago2‐RIP experiment revealed that miR‐2355‐5p was the highest enriched miRNAs among all miRNAs in ESCC cells harbouring WDFY3‐AS2 overexpression, compared to the pcDNA3.1 group (Figure S3), suggesting that WDFY3‐AS2 may bind to miR‐2355‐5p through the Ago2‐dependent pathway (Figure 5E). Further RIP experiment revealed that WDFY3‐AS2 and miR‐2355‐5p levels precipitated by anti‐Ago2 antibody were dramatically increased compared to IgG group (Figure 5F and G). In addition, Ago2‐RIP experiment showed that WDFY3‐AS2 enrichment in miR‐2355‐5p mimic group was markedly higher than that in NC group (Figure 5H). These data suggest that WDFY3‐AS2 and miR‐2355‐5p are in the same RNA‐induced silencing complex (RISC). To further verify whether WDFY3‐AS2 can interact with miR‐2355‐5p, the results of dual‐luciferase reporter system demonstrated that the luciferase intensity was significantly reduced by cotransfecting with miR‐2355‐5p mimic and WT‐WDFY3‐AS2 but not in the mutant vector without miR‐2355‐5p binding site in EC9706 and TE1 cells (Figure 5I and J). Meanwhile, WDFY3‐AS2 silencing obviously enhanced miR‐2355‐5p level, whereas WDFY3‐AS2 overexpression evidently decreased miR‐2355‐5p level in EC9706 and TE1 cells (Figure 5K and L). These data suggest that WDFY3‐AS2 directly regulates miR‐2355‐5p level in ESCC cells.

### 3.5 MiR‐2355‐5p promotes ESCC proliferation and invasion by targeting SOCS2/JAK2/Stat5 signalling pathway

**Figure 6:**
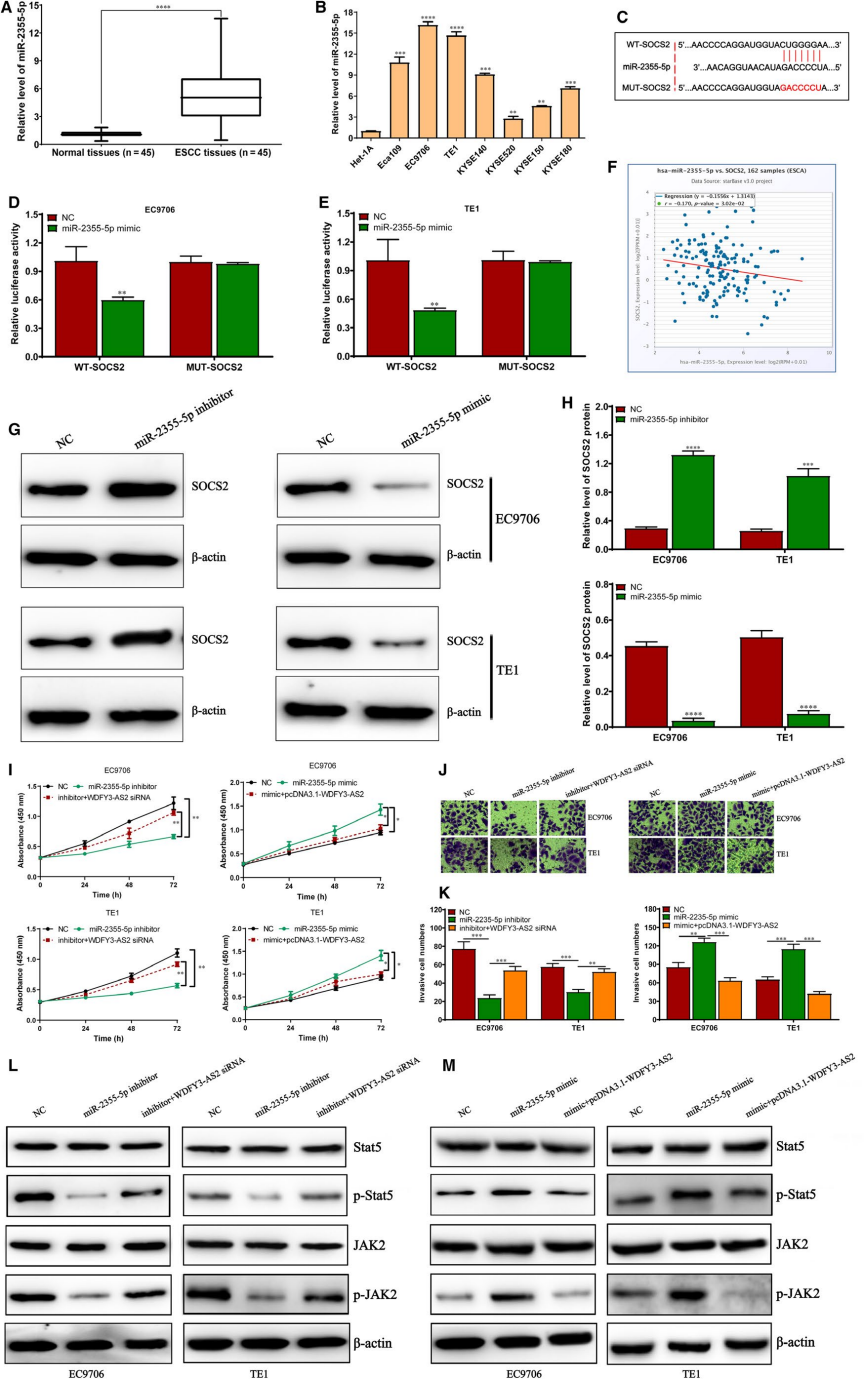
MiR‐2355‐5p promotes ESCC proliferation and invasion by targeting SOCS2/JAK2/Stat5 signalling pathway. A. qRT‐PCR assay for miR‐2355‐5p level in 45 cases of ESCC tissues and paired normal oesophageal epithelial tissues; B. qRT‐PCR assay for miR‐2355‐5p level in a panel of ESCC cells (Eca109, EC9706, TE1, KYSE140, KYSE520, KYSE150 and KYSE180) and normal oesophageal epithelial cell Het‐1A; C. The predicted miR‐2355‐5p binding sites in the SOCS2 3’UTR region; D and E. The luciferase activity assay in EC9706 and TE1 cells cotransfected with wide‐type (WT) or mutant SOCS2 plasmid together with miR‐2355‐5p mimic or NC; F. ENCORI Pan‐Cancer Analysis Platform was performed to analyse the correlation of miR‐2355‐5p and SOCS2 in 162 cases of ESCA patients; G. Western blot assay for SOCS2 protein expression after transfection with miR‐2355‐5p inhibitor or mimic; H. Relative level of SOCS2 protein after transfection with miR‐2355‐5p inhibitor or mimic; I. CCK‐8 assay for cell proliferation after transfection with miR‐2355‐5p inhibitor or mimic together with WDFY3‐AS2 siRNA or pcDNA3.1‐WDFY3‐AS2; J. Transwell experiment assay for cell invasion after transfection with miR‐2355‐5p inhibitor or mimic together with WDFY3‐AS2 siRNA or pcDNA3.1‐WDFY3‐AS2; K. Statistical assay of invasive cell numbers after transfection with miR‐2355‐5p inhibitor or mimic together with WDFY3‐AS2 siRNA or pcDNA3.1‐WDFY3‐AS2; L. Western blot assay for Stat5, p‐Stat5, JAK2 and p‐JAK2 protein expressions in NC group, miR‐2355‐5p inhibitor group and inhibitor plus WDFY3‐AS2 siRNA group; M. Western blot assay for Stat5, p‐Stat5, JAK2 and p‐JAK2 protein expression in NC group, miR‐2355‐5p mimic group and mimic plus pcDNA3.1‐WDFY3‐AS2 group. *P < .05, **P < .01, ***P < .001 and ****P < .0001 were considered as statistical significance. All data were obtained from three independent repeats

To verify the potential role of miR‐2355‐5p in ESCC, we firstly detected the miR‐2355‐5p expression in ESCC tissues and investigated the correlation between its expression and clinicopathological features. We found that miR‐2355‐5p expression was much higher in ESCC tissues and cells than that in normal oesophageal tissues and cell Het‐1A (Figure 6A and B), and its expression is tightly associated with TNM stage and lymph node metastasis (Table S5), suggesting miR‐2355‐5p may participate in ESCC development and progression. To further seek for the downstream regulatory axis of WDFY3‐AS2/miR‐2355‐5p in ESCC cells, TargetScan was used to predict the potential target genes of miR‐2355‐5p. Among all the predicted target genes, SOCS2 was confirmed to be a direct target of miR‐2355‐5p in EC9706 and TE1 cells (Figure 6C‐E). StarBase assay revealed that miR‐2355‐5p and SOCS2 expressions displayed a significant negative correlation (Figure 6F), which was verified in 45 cases of ESCC samples (Figure S4). To further confirm the possible role of miR‐2355‐5p in ESCC, miR‐2355‐5p inhibitor or mimic together with negative control was transfected into EC9706 and TE1 cells, as expected, miR‐2355‐5p inhibitor significantly suppressed miR‐2355‐5p level in EC9706 and TE1 cells, whereas miR‐2355‐5p mimic markedly promoted miR‐2355‐5p expression in EC9706 and TE1 cells (Figure S5). Further investigation demonstrated that miR‐2355‐5p inhibitor significantly increased SOCS2 protein level, whereas miR‐2355‐5p mimic dramatically suppressed SOCS2 protein level in EC9706 and TE1 cells (Figure 6G and H). Because of the direct interaction of WDFY3‐AS2 and miR‐2355‐5p, we further investigate the regulatory role of miR‐2355‐5p in cell proliferation and invasion by CCK‐8 and Transwell experiment. The results demonstrated that miR‐2355‐5p inhibitor significantly suppressed cell proliferation and invasion, but the efficacy was reversed by WDFY3‐AS2 siRNA. Conversely, miR‐2355‐5p mimic markedly promoted cell proliferation and invasion, and WDFY3‐AS2 overexpression dramatically reduced the promotive effects of miR‐2355‐5p mimic on EC9706 and TE1 cell proliferation and invasion (Figure 6I‐K), suggesting that WDFY3‐AS2/miR‐2355‐5p regulatory axis plays an important role in cell proliferation and invasion of ESCC cells. Recent report has demonstrated that SOCS family especially for SOCS2 has been regarded as the essential negative regulator of JAK/STAT signalling pathway.^30^ To further unveil the possible mechanisms mediated by WDFY3‐AS2/miR‐2355‐5p regulatory axis, we detected JAK2, p‐JAK2, Stat5 and p‐Stat5 level. The current findings demonstrated that miR‐2355‐5p inhibitor significantly reduced the levels of p‐Stat5 and p‐JAK2, and WDFY3‐AS2 siRNA reversed the suppressive effect mediated by miR‐2355‐5p inhibitor (Figure 6L). In contrast, miR‐2355‐5p mimic evidently increased the levels of p‐Stat5 and p‐JAK2, and WDFY3‐AS2 overexpression reversed the promotive effect elicited by miR‐2355‐5p mimic (Figure 6M). These data indicate that WDFY3‐AS2 sponges miR‐2355‐5p to up‐regulate SOCS2 expression and further results in the inactivation of JAK2/Stat5 signalling pathway in ESCC.

### 3.6 WDFY3‐AS2 functions a synergetic regulator with SOCS2 in ESCC cells by targeting JAK2/Stat5 signalling pathway

**Figure 7:**
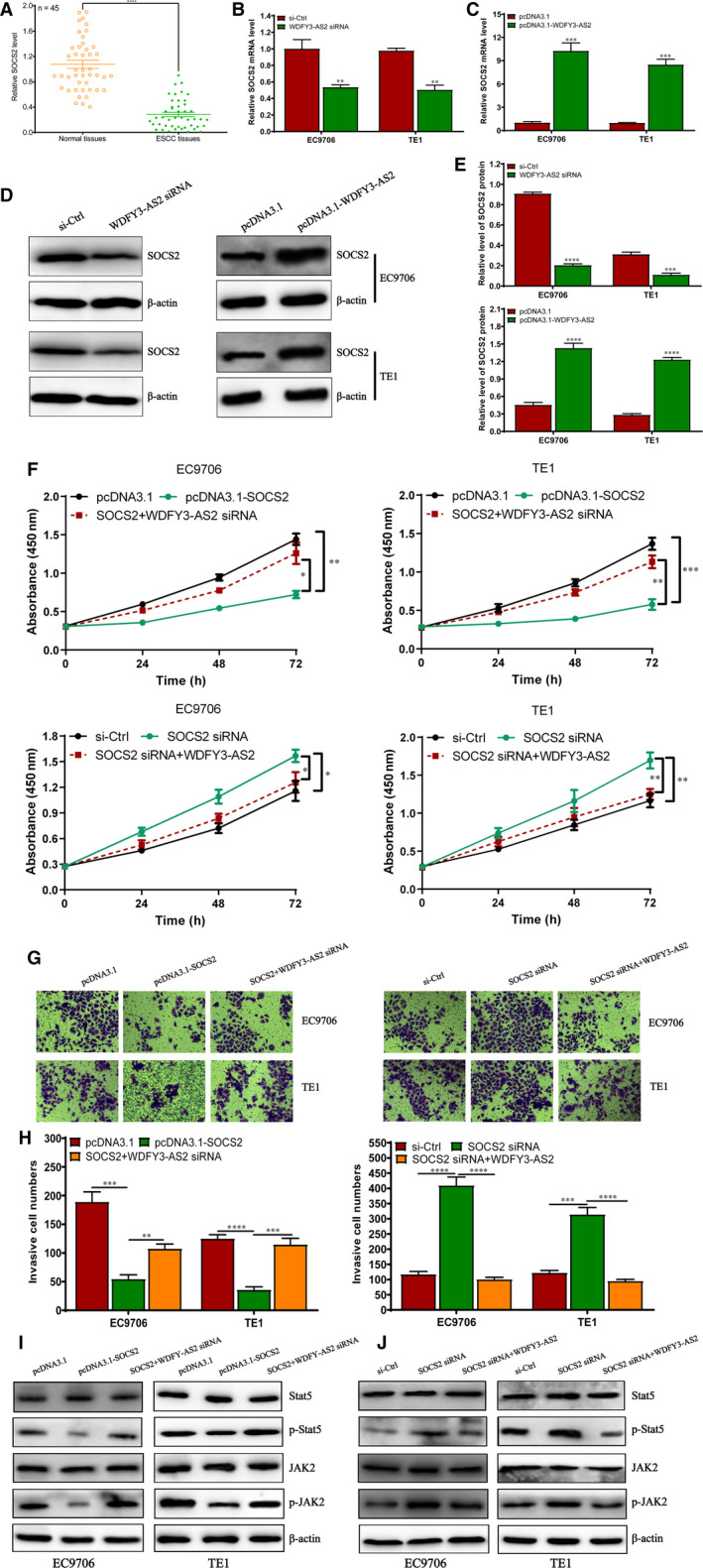
WDFY3‐AS2 is responsible for SOCS2‐mediated proliferation and invasion by targeting JAK2/Stat5 signalling pathway. A. qRT‐PCR detection for SOCS2 mRNA level in 45 cases of normal oesophageal epithelial tissues and ESCC tissues; B. The down‐regulation of WDFY3‐AS2 suppressed SOCS2 mRNA level in ESCC cells; C. WDFY3‐AS2 overexpression promoted SOCS2 mRNA level in ESCC cells; D. The effects of WDFY3‐AS2 level alteration on SOCS2 protein expression in ESCC cells; E. The relative level of SOCS2 protein in ESCC cells after transfection with si‐Ctrl, WDFY3‐AS2 siRNA, pcDNA3.1 and pcDNA3.1‐WDFY3‐AS2; F. WDFY3‐AS2 siRNA reverses cell proliferation inhibition mediated SOCS2 overexpression, whereas WDFY3‐AS2 overexpression reverses cell proliferation promotion triggered by SOCS2 siRNA; G. WDFY3‐AS2 siRNA reverses cell invasion inhibition mediated SOCS2 overexpression, whereas WDFY3‐AS2 overexpression reverses cell invasion promotion triggered by SOCS2 siRNA; H. Statistical assay of invasive cell numbers in different treatment ESCC cells; I. WDFY3‐AS2 siRNA reverses the suppression of JAK2/Stat5 signalling pathway elicited by SOCS2 overexpression in ESCC cells; J. WDFY3‐AS2 overexpression reverses the promotion of JAK2/Stat5 signalling pathway mediated by SOCS2 down‐regulation. *P < .05, **P < .01, ***P < .001 and ****P < .0001 were considered as statistical significance. All data were obtained from three independent repeats

**Figure 8:**
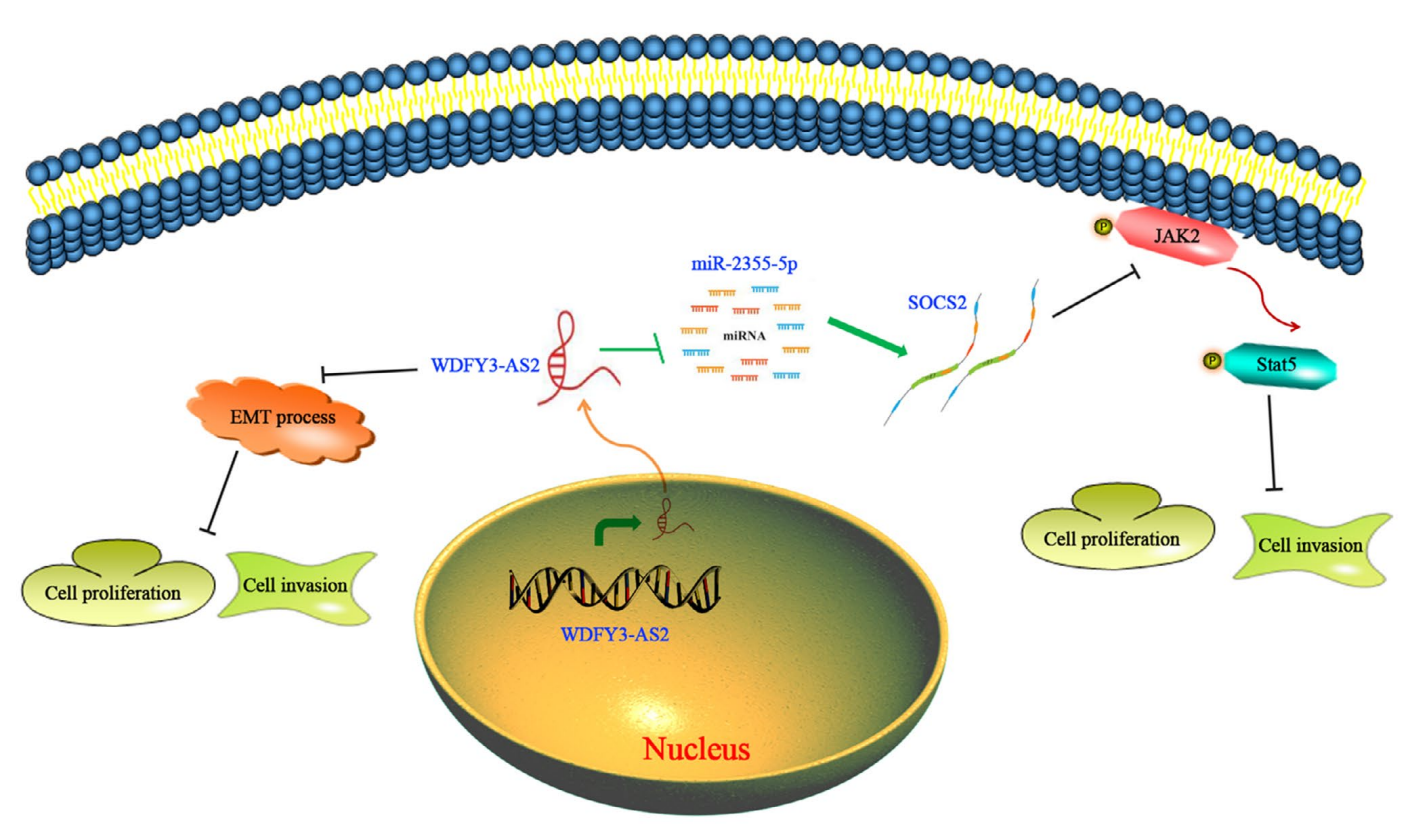
WDFY3‐AS2 contributes to the suppression of cell proliferation and invasion by manipulating miR‐2355‐5p/SOCS2 signalling axis. WDFY3‐AS2 displays the low expression in ESCC, its expression triggers the decrease of miR‐2355‐5p level, and further increases SOCS2 expression in ESCC cells, which will result in the inhibition of JAK2/Stat5 signalling pathway

To further investigate the role of SOCS2 in ESCC, We firstly detected SOCS2 expression in 45 cases of ESCC tissues and paired normal oesophageal tissues. We found that SOCS2 expression was much lower in ESCC tissues than that in normal oesophageal tissues (Figure 7A), and its expression exhibited marked positive correlation with WDFY3‐AS2 expression in ESCC tissues (Figure S6). In addition, SOCS2 expression was associated with TNM stage and lymph node metastasis (Table S6), suggesting that SOCS2 may be implicated in ESCC development and progression. Further investigation revealed that WDFY3‐AS2 siRNA reduced SOCS2 mRNA and protein expressions, whereas WDFY3‐AS2 overexpression promoted SOCS2 mRNA and protein expressions (Figure 7B‐E). To further verify the biological functions of SOCS2 in ESCC, we performed CCK‐8 and Transwell experiment for cell proliferation and invasion. The current results indicated that SOCS2 overexpression extremely suppressed cell proliferation and invasion, but the efficacy was reversed by WDFY3‐AS2 siRNA. Conversely, SOCS2 siRNA markedly promoted cell proliferation and invasion, and WDFY3‐AS2 overexpression dramatically reduced the promotive effects of SOCS2 siRNA on EC9706 and TE1 cell proliferation and invasion (Figure 7F‐H), suggesting that WDFY3‐AS2/SOCS2 regulatory axis plays an important role in cell proliferation and invasion of ESCC cells. Moreover, SOCS2 overexpession markedly suppressed the levels of p‐Stat5 and p‐JAK2 protein in EC9706 and TE1 cells, but the efficacy was reversed by WDFY3‐AS2 siRNA (Figure 7I). Conversely, SOCS2 siRNA had the opposite effects on the levels of p‐Stat5 and p‐JAK2 proteins (Figure 7J). Overall, these findings suggest that WDFY3‐AS2 is tumour suppressor lncRNA that inhibits cell proliferation and invasion by WDFY3‐AS2/miR‐2355‐5p/SOCS2/JAK2/Stat5 signalling pathway in ESCC cells (Figure 8).

## 4 DISCUSSION

The key findings of the current study revealed an essential prognostic factor WDFY3‐AS2, which played a pivotal role in ESCC development and progression. The current study demonstrated that low WDFY3‐AS2 expression was tightly associated with TNM stage, lymph node metastasis and poor prognosis of ESCC patients. WDFY3‐AS2 down‐regulation promoted cell proliferation and invasion, whereas WDFY3‐AS2 up‐regulation markedly suppressed cell proliferation and invasion in ESCC EC9706 and TE1 cells, coupled with EMT phenotype alterations. Mechanistically, WDFY3‐AS2 promoted SOCS2 expression by sponging miR‐2355‐5p and further triggered the inactivation of JAK2/Stat5 signalling pathway, which resulted in the suppression of cell proliferation and invasion in ESCC cells. Overall, these findings reveal the clinical significance of WDFY3‐AS2 and the essential roles of WDFY3‐AS2/miR‐2355‐5p/SOCS2 signalling axis in ESCC development and progression.

Increasing evidence has demonstrated that lncRNAs widely participate in tumour development and progression, and may be an underlying prognostic factor for multiple different tumour types. Jiang X, et al reported that linc01234 level was markedly increased in colorectal cancer (CRC) tissues, and its expression was associated with tumour stage, tumour size, metastasis and reduced overall survival of the patients with CRC.^31^ ZEB1‐AS1 overexpression was related to TNM stage and lymph node metastasis as well as poor prognosis in ESCC, and its level may be a novel prognostic factor for ESCC patients.^22^ In addition, WDFY3‐AS2 was independently related to overall survival (OS) of patients, and patients with high WDFY3‐AS2 expression had longer OS than the low expression ones in diffuse glioma.^21^ To verify the potential roles of WDFY3‐AS2 in ESCC progression and development, GEO DataSets, TCGA database and qRT‐PCR were used to detect the expression of WDFY3‐AS2 in ESCA tissues and cells. Our results demonstrated that WDFY3‐AS2 was dramatically down‐regulated in ESCA tissues by GEO DataSets and TCGA database, which was further confirmed by qRT‐PCR in 45 cases of ESCC tissues and paired normal oesophageal epithelial tissues. Furthermore, WDFY3‐AS2 expression was tightly associated with TNM stage and lymph node metastasis, suggesting that WDFY3‐AS2 may participate in ESCC progression. Most notably, the survival time of the patients harbouring low WDFY3‐AS2 level was significantly shorter than those with high WDFY3‐AS2 level. Our findings suggest that WDFY3‐AS2 plays an important role in ESCC development and progression, and may be a key prognostic factor for ESCC patients.

Tumour proliferation and invasion and metastasis are two main biological hallmarks of tumours,^32^ which will be the important phenotypes for tumour investigation. In the current study, we found that WDFY3‐AS2 down‐regulation promoted cell proliferation and invasion, whereas WDFY3‐AS2 up‐regulation markedly suppressed cell proliferation and invasion in ESCC EC9706 and TE1 cells, coupled with EMT phenotype alterations, implying targeting WDFY3‐AS2 may be a novel therapeutic strategy for ESCC patients. Previous report revealed that lncRNA functions are tightly associated with its subcellular localization. In general, nuclear lncRNAs play the important regulatory in chromatin structure and gene transcription,^33^, ^34^ whereas cytoplasmic lncRNAs function as ceRNA to regulate gene expression.^35^ Given the subcellular localization of WDFY3‐AS2 in ESCC cytoplasm by online software lncATLAS prediction, qRT‐PCR and FISH assay, we predicted the possible binding miRNAs by the DIANA Tools, and the top 25 miRNAs were selected for further assay. Ago2‐RIP experiment revealed that miR‐2355‐5p was the highest enriched miRNAs in all investigated miRNAs in ESCC cells when WDFY3‐AS2 was overexpressed, and further investigation revealed that miR‐2355‐5p was a direct target of WDFY3‐AS2 in ESCC cells. These findings suggest that WDFY3‐AS2/miR‐2355‐5p may be an important regulatory axis in ESCC development and progression.

Furthermore, miRNAs, a kind of non‐coding RNA molecules, have been verified to function as negative regulators of expressions of target genes by binding to the 3’‐untranslated region of candidate mRNAs, further manipulating tumour development and progression.^36^ MiR‐2355‐5p negatively manipulated ERRFI1 expression and blocked the suppressive effects of ERRFI1 on cell proliferation and inflammation in nucleus pulposus (NP) cells.^37^ In the current study, miR‐2355‐5p was up‐regulated in ESCC tissues and cells, and its expression was negatively correlated with WDFY3‐AS2 expression in ESCC tissues. MiR‐2355‐5p significantly accelerated ESCC cell proliferation and invasion, whereas miR‐2355‐5p down‐regulation suppressed ESCC cell proliferation and invasion; however, these phenotypes were markedly reversed by WDFY3‐AS2 up‐regulation or down‐regulation. To further the possible mechanism of miR‐2355‐5p in ESCC, the potential target gene of miR‐2355‐5p in ESCC was predicted by TargetScan, SOCS2 was selected for further investigation. As expected, dual‐flurecase reporter assay system verified that SOCS2 was a direct target gene of miR‐2355‐5p in ESCC cells, miR‐2355‐5p inhibitor significantly enhanced SOCS2 mRNA and protein levels, whereas miR‐2355‐5p mimic markedly reduced the expressions of SOCS2 mRNA and protein. Several studies have demonstrated that SOCS family proteins are modulators of cytokine and growth factor signalling whose aberrant regulation has been linked to a variety of inflammatory and neoplastic diseases, and its family members including SOCS1, SOCS2 and SOCS3 were verified to be important negative regulator of JAK/Stat signalling pathway, especially for SOCS2.^38^ In addition, SOCS2 has been investigated as molecular targets of many microRNAs in multiple different tumours, including miR‐196a/miR‐196b,^39^ miR‐492^40^ and miR‐875,^41^ suggesting that SOCS2 may form an important regulatory network mediated by miRNAs. To verify the regulatory role of miR‐2355‐5p on JAK2/Stat5 signalling pathway in ESCC, we further detected the expressions of p‐JAK2 and p‐Stat5 in ESCC cells. We found that miR‐2355‐5p inhibitor dramatically suppressed the levels of p‐JAK2 and p‐Stat5 proteins, but miR‐2355‐5p mimic extremely promoted the expressions of p‐JAK2 and p‐Stat5 in ESCC cells; however, the effects were reversed by WDFY3‐AS2 down‐regulation or overexpression. These findings indicate that WDFY3‐AS2 plays an important regulatory role in cell proliferation and invasion mediated by miR‐2355‐5p in ESCC.

In the last few years, SOCS proteins have been considered as potential tumour suppressors in many different tumours. Loss of SOCS2 in breast cancer resulted in a growth‐promoting effect.^42^, ^43^ Reduced SOCS2 level is markedly related to advanced TNM stage and appears to be a prognostic marker in hepatocellular carcinoma.^44^ To further explore the biological role of SOCS2 in development and progression, SOCS2 down‐regulation was found in this study, and SOCS2 expression was tightly associated with TNM stage and lymph node metastasis of ESCC patients. Because of the function of lncRNAs as a ceRNA linking to the target gene of miRNAs, we found WDFY3‐AS2 siRNA significantly suppressed SOCS2 expression, but WDFY3‐AS2 overexpression markedly promoted SOCS2 expression in ESCC cells. Further investigation revealed that SOCS2 overexpression suppressed cell proliferation and invasion, whereas SOCS2 down‐regulation promoted cell proliferation and invasion, which was reversed by WDFY3‐AS2 siRNA or overexpression, respectively. Mechanistically, SOCS2 overexpression obviously suppressed the levels of p‐JAK2 and p‐Stat5 proteins, but SOCS2 down‐regulation evidently promoted the expressions of p‐JAK2 and p‐Stat5 in ESCC cells; however, the effects were reversed by WDFY3‐AS2 down‐regulation or overexpression. These findings imply that WDFY3‐AS2 functions as a key regulator in cell proliferation and invasion by targeting miR‐2355‐5p/SOCS2/JAK2/Stat5 signalling pathway in ESCC.

In conclusion, the current data presented herein suggest that WDFY3‐AS2 might be a novel predictor for ESCC patients’ prognosis. WDFY3‐AS2 suppressed ESCC proliferation and invasion by sponging miR‐2355‐5p to up‐regulate SOCS2 expression, further resulting in inactivation of JAK2/Stat5 signalling pathway, which will trigger the suppression of cell proliferation and invasion in ESCC cells. These findings indicate that WDFY3‐AS2 plays an important role in ESCC development and progression, and may be an underlying prognostic factor and promising therapeutic target in ESCC.

## CONFLICT OF INTEREST

The authors declare that they have no competing interests.

## AUTHOR CONTRIBUTION

**Qing Zhang:** Data curation (equal); Investigation (equal). **Fangxia Guan:** Data curation (equal); Investigation (equal). **Tianli Fan:** Data curation (equal); Investigation (equal). **Shenglei Li:** Investigation (supporting); Methodology (lead). **Shanshan Ma:** Investigation (supporting); Writing‐review & editing (supporting). **Yanting Zhang:** Investigation (supporting); Writing‐review & editing (supporting). **Wenna Guo:** Validation (supporting). **Hongtao Liu:** Funding acquisition (lead); Project administration (lead); Supervision (lead); Writing‐original draft (lead).

## ETHICS APPROVAL AND CONSENT TO PARTICIPATE

This study was reviewed and approved by the Research and Ethics Committee of the First Affiliated Hospital of Zhengzhou University (Zhengzhou, China). The study was conduscted in accordance with the International Ethical Guidelines for Biomedical Research Involving Human Subjects. All patients provided informed consent to participate in the study.

## Supporting information

Supplementary Material

Table S1‐S6

## Data Availability

The data that support the findings of this study are available from the corresponding author upon reasonable request.
