# Supplementary material for: LncRNA WDFY3‐AS2 suppresses proliferation and invasion in oesophageal squamous cell carcinoma by regulating miR‐2355‐5p/SOCS2 axis
Source: J Cell Mol Med. 2020 Jun 14;24(14):8206–20. doi: 10.1111/jcmm.15488 (PMC7348145; doi:10.1111/jcmm.15488)
Supplement: Supplementary file 1 — Supplementary Material [file JCMM-24-8206-s001.docx]

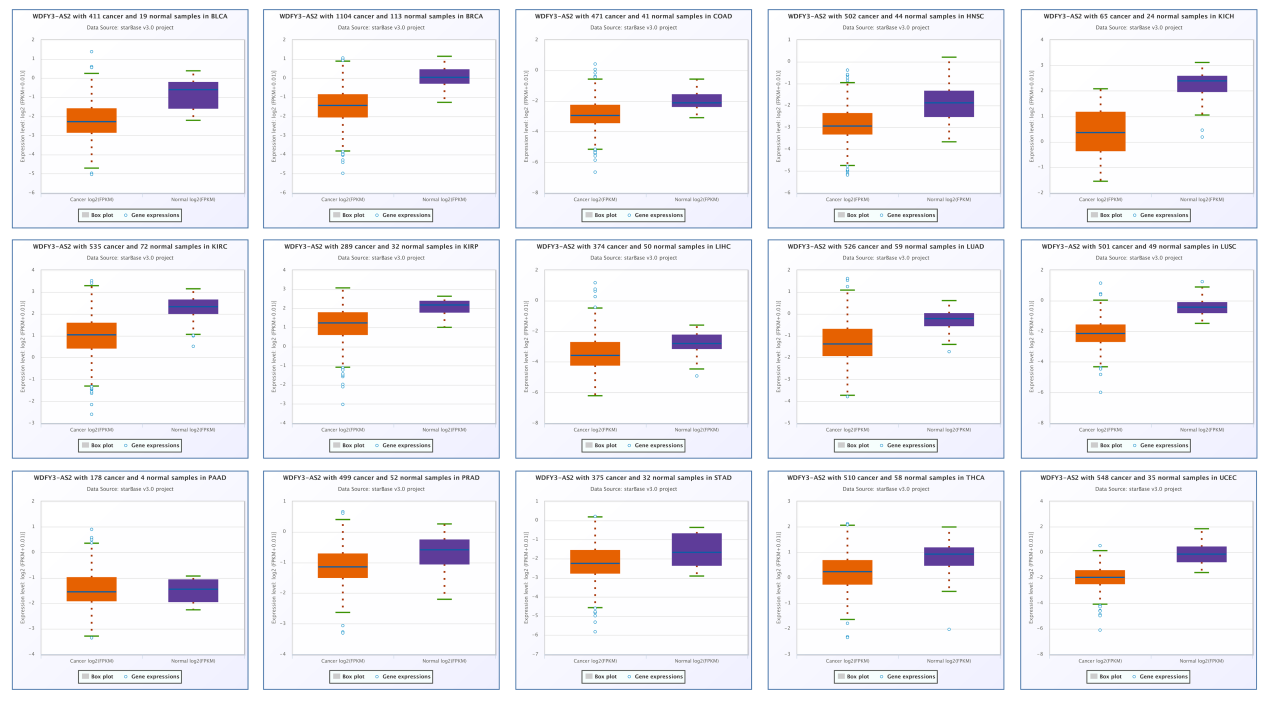


Supplementary Figure 1


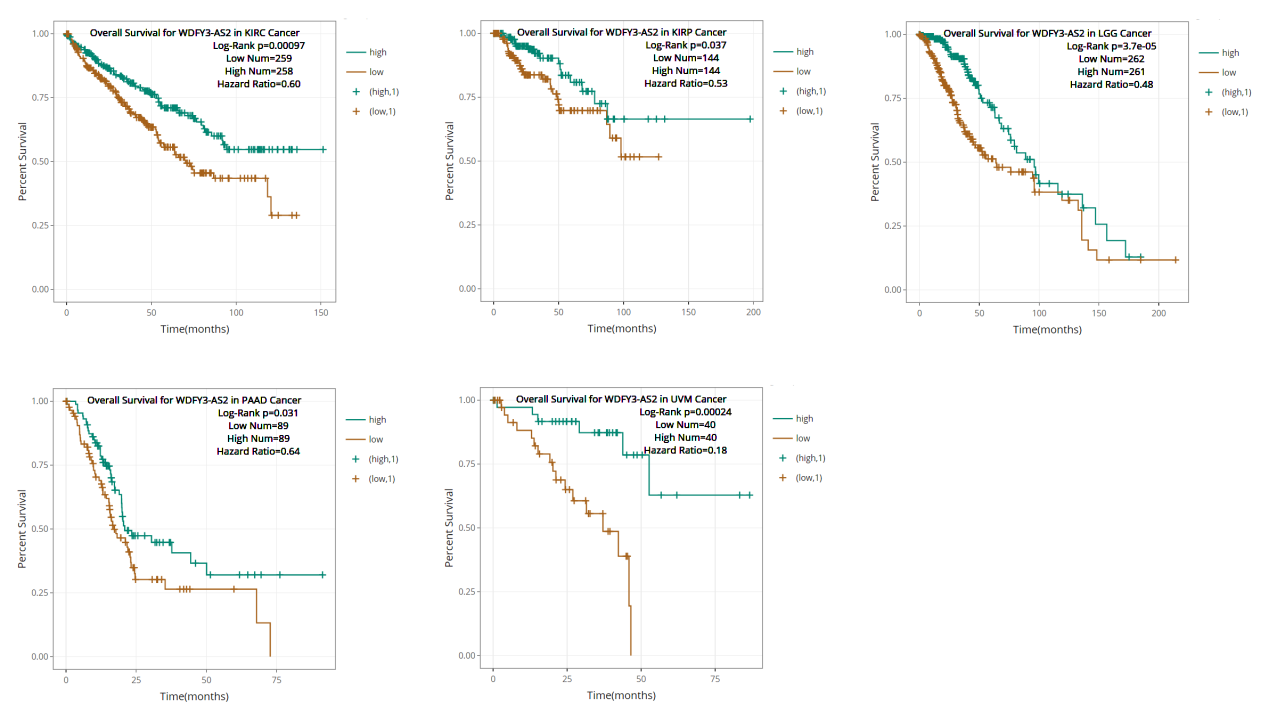


Supplementary Figure 2


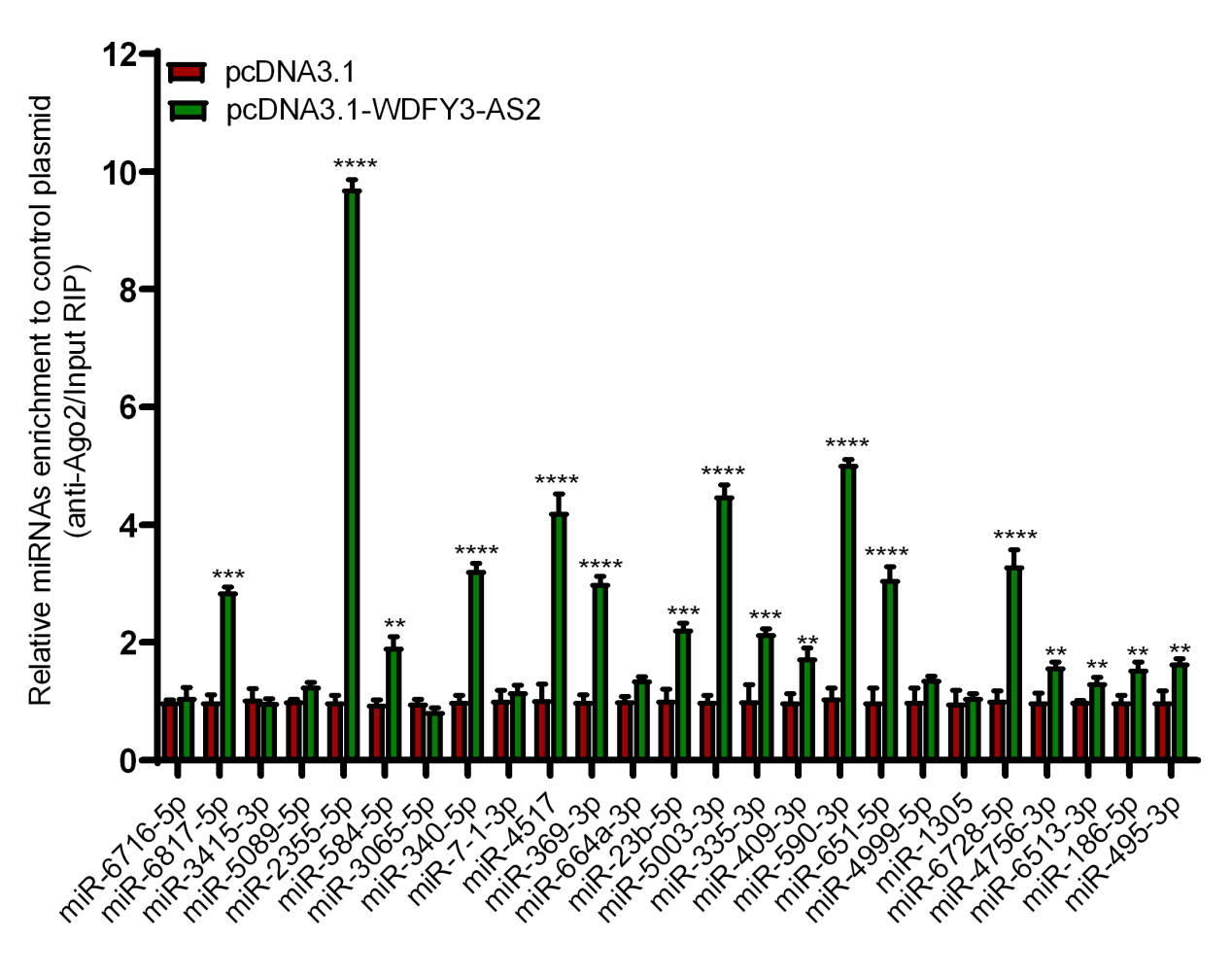


Supplementary Figure 3


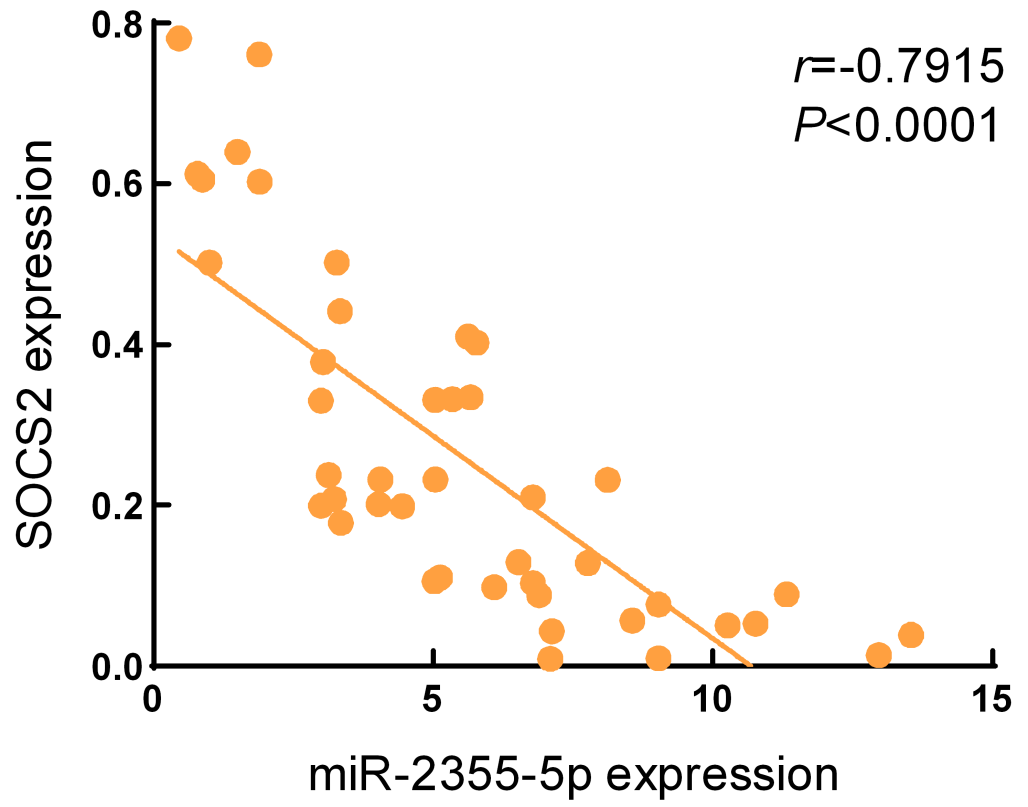


Supplementary Figure 4


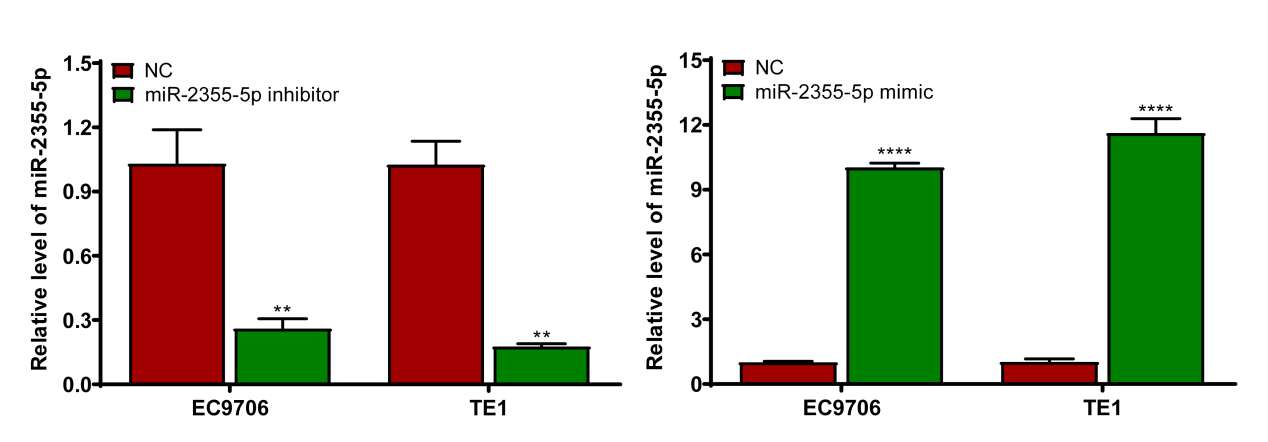


Supplementary Figure 5


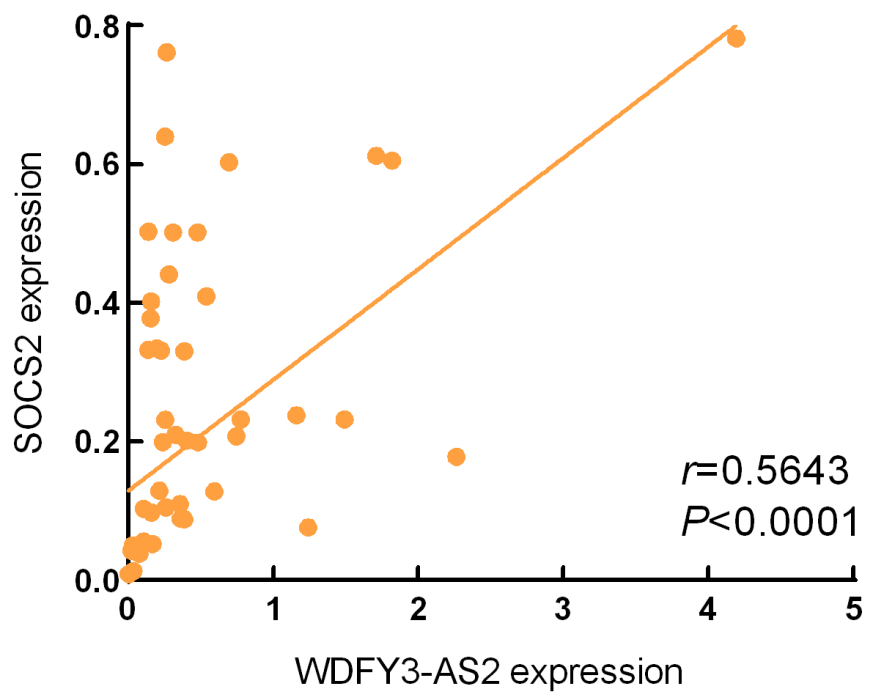


Supplementary Figure 6
