# Supplementary material for: LncRNA WDFY3‐AS2 suppresses proliferation and invasion in oesophageal squamous cell carcinoma by regulating miR‐2355‐5p/SOCS2 axis
Source: J Cell Mol Med. 2020 Jun 14;24(14):8206–20. doi: 10.1111/jcmm.15488 (PMC7348145; doi:10.1111/jcmm.15488)
Supplement: Supplementary file 2 — Table S1‐S6 [file JCMM-24-8206-s002.docx]

**Supplementary Table 1. The sequences of WDFY3-AS2 siRNA and si-Ctrl used for synthesis**

| Name | Sequence (5’ to 3’) |
| --- | --- |
| Si-Ctrl |  |
| sense | UUCUCCGAACGUGUCACGUTT |
| antisense | ACGUGACACGUUCGGAGAATT |
| WDFY3-AS2 |  |
| WDFY3-AS2#1 sense | CCACAAACUUGAAUACCUUTT |
| WDFY3-AS2#1 antisense | AAGGUAUUCAAGUUUGUGGTT |
| WDFY3-AS2#2 sense | GCGUCAAGUUAAGGGCAUUTT |
| WDFY3-AS2#2 antisense | AAUGCCCUUAACUUGACGCTT |
| WDFY3-AS2#3 sense | CCUUAACCCUACACCCUUUTT |
| WDFY3-AS2#3 antisense | AAAGGGUGUAGGGUUAAGGTT |

**Supplementary Table 2. The primer sequence used for vector construction**

| Vector name | Sequence (5’ to 3’) |
| --- | --- |
| pcDNA3.1-WDFY3-AS2-F | CCCAAGCTTAGTACAAGAGAACTGGAGCA (*Hin*dIII) |
| pcDNA3.1-WDFY3-AS2-R | TGCTCTAGAGAAAGTATGTGCTGCTTTATTTTCATATT (*Xba*I) |
| pcDNA3.1-SOCS2-F | CGCGGATCCATGACCCTGCGGTGCCTTGA (*Bam*HI) |
| pcDNA3.1-SOCS2-R | TGCTCTAGATTATACCTGGAATTTATATTC (*Xba*I) |

**Supplementary Table 3. qRT-PCR primers used in this study**

| Gene | Sequence (5’ to 3’) |
| --- | --- |
| WDFY3-AS2-F | TTGGGGCACTCATCCCATTC |
| WDFY3-AS2-R | TTGAGCTCGGACTGTGCATT |
| U6-F | CTCGCTTCGGCAGCACA |
| U6-R | AACGCTTCACGAATTTGCGT |
| GAPDH-F | CGGAGTCAACGGATTTGGTCGTAT |
| GAPDH-R | AGCCTTCTCCATGGTGGTGAAGAC |
| miR-2355-5p | ATCCCCAGATACAATGGACAA |
| SOCS2-F | GCAAGGATAAGCGGACAGGT |
| SOCS2-R | GTTGGTAAAGGCAGTCCCCA |

**Supplementary Table 4. The predicted miRNAs that bind with WDFY3-AS2 according to DIANA Tools**

| Gene | miRNA | miTG-score |
| --- | --- | --- |
| ENSG00000180769(WDFY3-AS2) | miR-6716-5p | 0.995 |
| ENSG00000180769(WDFY3-AS2) | miR-6817-5p | 0.987 |
| ENSG00000180769(WDFY3-AS2) | miR-3415-3p | 0.984 |
| ENSG00000180769(WDFY3-AS2) | miR-5089-5p | 0.979 |
| ENSG00000180769(WDFY3-AS2) | miR-2355-5p | 0.971 |
| ENSG00000180769(WDFY3-AS2) | hsa-miR-584-5p | 0.951 |
| ENSG00000180769(WDFY3-AS2) | hsa-miR-3065-5p | 0.946 |
| ENSG00000180769(WDFY3-AS2) | hsa-miR-340-5p | 0.946 |
| ENSG00000180769(WDFY3-AS2) | hsa-miR-7-1-3p | 0.926 |
| ENSG00000180769(WDFY3-AS2) | hsa-miR-4517 | 0.920 |
| ENSG00000180769(WDFY3-AS2) | hsa-miR-369-3p | 0.913 |
| ENSG00000180769(WDFY3-AS2) | hsa-miR-664a-3p | 0.909 |
| ENSG00000180769(WDFY3-AS2) | hsa-miR-23b-5p | 0.905 |
| ENSG00000180769(WDFY3-AS2) | hsa-miR-5003-3p | 0.884 |
| ENSG00000180769(WDFY3-AS2) | hsa-miR-335-3p | 0.882 |
| ENSG00000180769(WDFY3-AS2) | hsa-miR-409-3p | 0.880 |
| ENSG00000180769(WDFY3-AS2) | hsa-miR-590-3p | 0.868 |
| ENSG00000180769(WDFY3-AS2) | hsa-miR-651-5p | 0.865 |
| ENSG00000180769(WDFY3-AS2) | hsa-miR-4999-5p | 0.861 |
| ENSG00000180769(WDFY3-AS2) | hsa-miR-1305 | 0.858 |
| ENSG00000180769(WDFY3-AS2) | hsa-miR-6728-5p | 0.855 |
| ENSG00000180769(WDFY3-AS2) | hsa-miR-4756-3p | 0.853 |
| ENSG00000180769(WDFY3-AS2) | hsa-miR-6513-3p | 0.852 |
| ENSG00000180769(WDFY3-AS2) | hsa-miR-186-5p | 0.851 |
| ENSG00000180769(WDFY3-AS2) | has-miR-495-3p | 0.843 |

**Supplementary Table 5. The associations of miR-2355-5p expression with clinicopathological features in ESCC**

| Features | n | miR-2355-5p expression | | *X^2^* | *P* value |
| --- | --- | --- | --- | --- | --- |
|  |  | + | - |  |  |
| Gender |  |  |  |  |  |
| Male | 28 | 16 | 12 | 0.432 | 0.511 |
| Female | 17 | 8 | 9 |  |  |
| Age (years) |  |  |  |  |  |
| ≤60 | 16 | 10 | 6 |  |  |
| >60 | 29 | 14 | 15 | 0.838 | 0.360 |
| Invasive depth |  |  |  |  |  |
| Superficial layer | 5 | 2 | 3 |  |  |
| Deep layer | 40 | 22 | 18 | 0.402 | 0.526 |
| Differentiation degree |  |  |  |  |  |
| High/moderate | 34 | 17 | 17 | 0.621 | 0.431 |
| Poor | 11 | 7 | 4 |  |  |
| TNM stage |  |  |  |  |  |
| I-II | 27 | 9 | 18 |  |  |
| III-IV | 18 | 15 | 3 | 10.848 | 0.001 |
| Lymph node metastasis |  |  |  |  |  |
| Yes | 21 | 16 | 5 | 8.265 | 0.004 |
| No | 24 | 8 | 16 |  |  |

**Supplementary Table 6. The correlation between SOCS2 expression and clinicopathological features in ESCC**

| Features | n | SOCS2 expression | | *X^2^* | *P* value |
| --- | --- | --- | --- | --- | --- |
|  |  | + | - |  |  |
| Gender |  |  |  |  |  |
| Male | 28 | 6 | 22 | 2.008 | 0.156 |
| Female | 17 | 7 | 10 |  |  |
| Age (years) |  |  |  |  |  |
| ≤60 | 16 | 5 | 11 |  |  |
| >60 | 29 | 8 | 21 | 0.067 | 0.795 |
| Invasive depth |  |  |  |  |  |
| Superficial layer | 5 | 2 | 3 |  |  |
| Deep layer | 40 | 11 | 29 | 0.338 | 0.561 |
| Differentiation degree |  |  |  |  |  |
| High/moderate | 34 | 11 | 23 | 0.812 | 0.367 |
| Poor | 11 | 2 | 9 |  |  |
| TNM stage |  |  |  |  |  |
| I-II | 27 | 11 | 16 |  |  |
| III-IV | 18 | 2 | 16 | 4.615 | 0.032 |
| Lymph node metastasis |  |  |  |  |  |
| Yes | 21 | 3 | 18 | 4.087 | 0.043 |
| No | 24 | 10 | 14 |  |  |
